# *Aspergillus* Tracheobronchitis With Mediastinal Lymphadenopathy in a Patient With Well-Controlled HIV Infection

**DOI:** 10.1155/crdi/9748358

**Published:** 2024-11-26

**Authors:** Ekachai Singhatiraj, Korsin Tiengburanatarm, Krit Pongpirul

**Affiliations:** ^1^Department of Medicine, Bumrungrad International Hospital, Bangkok, Thailand; ^2^Clinical Research Center, Bumrungrad International Hospital, Bangkok, Thailand; ^3^Center of Excellence in Preventive and Integrative Medicine, Faculty of Medicine, Chulalongkorn University, Bangkok, Thailand; ^4^Department of International Health, Johns Hopkins Bloomberg School of Public Health, Baltimore, Maryland, USA; ^5^Department of Infection Biology & Microbiomes, Faculty of Health and Life Sciences, University of Liverpool, Liverpool, UK

**Keywords:** antiretroviral therapy, *Aspergillus* tracheobronchitis, hemoptysis, HIV infection, invasive pulmonary aspergillosis, opportunistic infections, voriconazole

## Abstract

**Background:** *Aspergillus* tracheobronchitis (AT) is an uncommon yet severe form of invasive pulmonary aspergillosis, with a notably low incidence among individuals living with HIV infection—accounting for merely 4.5% (7 out of 156 cases) in recent reviews. The advent of modern antiretroviral therapy (ART) has significantly altered the landscape of opportunistic infections in HIV, rendering conditions like AT rare in well-controlled cases.

**Case Presentation:** We present the case of a woman in her mid-20s with well-managed HIV infection who experienced a 4-week history of fever and dyspnea. Diagnostic procedures, including bronchoscopy, revealed granulation tissue obstructing her right main bronchus. Cultures confirmed infection with *Aspergillus fumigatus*, leading to a diagnosis of AT. Despite initial positive response to voriconazole treatment, the patient developed severe hemoptysis and unfortunately succumbed to the complication.

**Conclusion:** This case underscores the critical need for healthcare providers to consider AT in the differential diagnosis of respiratory symptoms in HIV-positive patients, even when HIV is well-controlled with ART. Early recognition and prompt antifungal therapy are essential for improving outcomes. Clinicians should remain vigilant for severe complications like hemoptysis, which can occur despite appropriate therapy. This report highlights the ongoing necessity for vigilance and proactive intervention in the care of individuals living with HIV.

**Summary**

• *Aspergillus* tracheobronchitis is an uncommon presentation in individuals who have their HIV infection effectively managed.
• In immunocompromised individuals, the presence of symptoms such as cough, chest pain, fever, dyspnea, and signs of upper airway obstruction should prompt consideration of *Aspergillus* tracheobronchitis.
• There's a notable trend of *Aspergillus* tracheobronchitis diagnoses among lung transplant recipients, especially within the initial 3 months post-transplantation.
• The diagnosis hinges on bronchoscopic visualization; indicators include erythema, pseudomembranous formations, or ulcerations. This is further solidified by histopathological and/or positive fungal culture results.
• Upon diagnosis, timely initiation of treatment with voriconazole is crucial for optimal outcomes.

## 1. Background

*Aspergillus* tracheobronchitis (AT) is an unusual form of invasive pulmonary aspergillosis (IPA), accounting for less than 10% of cases [1]. Primary predisposing conditions include hematologic malignancies, neutropenia, solid organ transplantation, chronic corticosteroids, and acquired immunodeficiency syndrome (AIDS). Diagnosis is usually delayed due to its nonspecific clinical presentation and the lack of radiographic findings in the early stages [2]. Early recognition is essential for proper diagnosis and treatment to reduce mortality [2].

Three forms of AT include (1) pseudomembranous tracheobronchitis (pervasive involvement of the entire tracheobronchial tree, with a membranous slough overlying the mucosa containing *Aspergillus*), (2) ulcerative AT (histological invasion of the abnormal area of the bronchial mucosa or cartilage showed hyphae consistent with *Aspergillus*), and (3) obstructive bronchial aspergillosis described in patients with AIDS and heart transplant (thick mucus plugs without evidence of invasion or allergic manifestations) [3]. Most patients with AT had a favorable prognosis with early diagnosis and effective antifungal treatment. The morphological characteristics of intraluminal lesions could have a prognostic value [4].

Invasive aspergillosis (IA) is an unusual opportunistic infection in a patient with HIV, but the incidence is unknown. In a large series of autopsies, aspergillosis was found in 1.4% of pulmonary infections in patients with HIV [5]. The overall prevalence of HIV infection in the AT case review was 4.5% (7/156 cases) [6], a figure that appears low relative to Thailand's broader HIV prevalence of 5,60,000 out of 71.6 million, as per WHO data.

## 2. Case Presentation

A woman in her mid-20s, previously diagnosed with well-controlled HIV infection and treated for pulmonary tuberculosis (TB), presented with a 4-week history of fever and dyspnea. Her HIV diagnosis dates back to 2020, and she has since maintained a regimen of ART (tenofovir, emtricitabine, and efavirenz). At our hospital, her CD4 count was 414 cells/mm^3^ (26.7%), and her HIV viral load was undetectable. Before visiting our institution, she had sought care at an outside hospital where a chest X-ray showed a right pleural effusion. Subsequent thoracocentesis at that hospital revealed a pleural fluid white blood cell count of 1600/*μ*L, with 87% being mononuclear cells. The total protein of the pleural fluid was 4.9 g/dL, in contrast to her serum total protein level of 9.2 g/dL, and her lactate dehydrogenase (LDH) was recorded at 166 IU/L. Given her previous TB history, the external facility empirically started her on antituberculosis medications, including isoniazid, rifampicin, and ethambutol. Pyrazinamide was omitted due to its associated side effects, particularly nausea. However, her condition did not improve on this regimen. It's worth noting that while her TB history might suggest a reactivation as the cause of her current symptoms, the lack of response to the empirical treatment raised questions. Regrettably, essential diagnostic tests such as adenosine deaminase, PCR for TB, and TB culture were not performed on the pleural fluid by the outside hospital. Upon examination in our facility, she was afebrile with decreased breath sound in her right lung. Blood tests indicated a CD4 lymphocyte count of 414 cells/mm^3^ (27%) and an undetectable HIV viral load. Importantly, she had been on ART (tenofovir, emtricitabine, and efavirenz) for 4 years and was not on a protease inhibitor (PI) regimen.

### 2.1. Investigations

**Figure 1:**
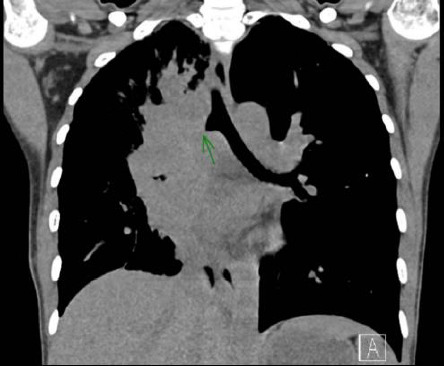
Chest CT. Chest CT showed extensive matted lymph nodes in the right hilar, paratracheal, subcarinal, interlobar, right supraclavicular, and right lower cervical regions with extensive peribronchovascular soft tissues, causing total right mainstem bronchial obstruction, obliteration of the segmental bronchi of RUL and RML.

**Figure 2:**
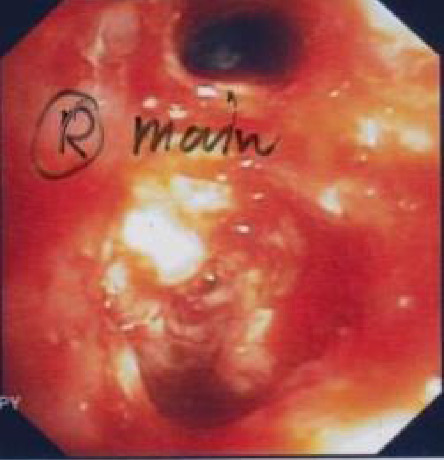
Bronchoscopy. Bronchoscopy revealed a widening of the carina, granulation tissue that obstructs the right main bronchus.

**Figure 3:**
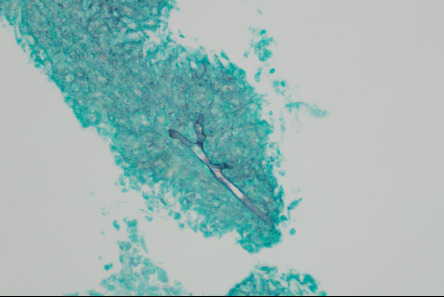
Tracheal biopsy. The Gomori methenamine silver stain (GMS stain) from the tracheal biopsy showed acute angle septate hyphae, which is consistent with *Aspergillus* species.

A chest CT scan was conducted, revealing a pattern of extensive lymphadenopathy. Affected regions included the right hilar, paratracheal, subcarinal, interlobar, right supraclavicular, and right lower cervical lymph nodes. The scan also depicted significant peribronchovascular soft tissue proliferation, leading to a complete obstruction of the right main bronchus and the obliteration of segmental bronchi in both the right upper lobe (RUL) and the right middle lobe (RML) as visualized in Figure 1. Subsequently, a bronchoscopy was performed, which demonstrated prominent granulation tissue obstructing within the right main bronchus. This can be further appreciated in Figure 2. For a more definitive diagnosis, a bronchial tissue biopsy was undertaken. Histopathological examination of the sample unveiled necrotizing inflammation suggestive of a granulomatous fungal infection. Gomori methenamine silver (GMS) stain further pinpointed the presence of acute angle septate hyphae, as shown in Figure 3. Complementary to the biopsy results, the bronchial washing fluid tested positive for the galactomannan (GM) antigen, recording an index level of 0.83. In line with the joint clinical guidelines by the European Society for Clinical Microbiology and Infectious Diseases, the European Confederation of Medical Mycology, and the European Respiratory Society (ESCMID-ECMM-ERS), we highlighted the enhanced sensitivity of GM detection in bronchoalveolar lavage (BAL) fluids for diagnosing IA compared to traditional culture methods [7]. The GM is reported as an optical density index (ODI) with its efficacy underscored as a diagnostic measure. Notably, an optimal cut-off for GM positivity lies between 0.5 and 1.0. Cultures from the sample subsequently identified the causative pathogen as the *Aspergillus fumigatus* complex.

### 2.2. Differential Diagnosis

Considering the patient's clinical presentation, a plethora of differential diagnoses emerged. Her prior treatment for TB in 2020 naturally postulates the possibility of a TB reactivation as the cause of her symptoms, including the development of pleural effusion. However, her nonresponsiveness to empiric antituberculosis therapy made this less probable. Though aspergillosis is not often placed at the forefront of differential diagnoses in patients with HIV, the finding from bronchial biopsy and the bronchial washing fluid, coupled with the pattern of extensive lymphadenopathy from the chest CT, made this fungal infection a prime contender. It is noteworthy to mention that manifestations such as pleural effusion and mediastinal lymphadenopathy associated with aspergillosis, especially *Aspergillus fumigatus* complex, are not extensively documented in the literature. Other infectious etiologies that could mimic this clinical picture include bacterial pneumonia and other fungal infections such as histoplasmosis and cryptococcosis. The patient's HIV status, despite being well-controlled, always poses an increased risk for opportunistic infections, thereby broadening the differential lists. Lastly, while the infectious causes loomed large, it is imperative to consider noninfectious etiologies. Malignancies, specifically lymphomas or primary lung cancers, given the extent of lymphadenopathy, should always be included in the differential spectrum, especially with such extensive lymphadenopathy. The investigative findings, including the CT scan patterns and bronchoscopy results, in conjunction with her well-documented clinical history, steered the diagnostic journey towards a more concrete conclusion, ultimately pinpointing the causative agent and the underlying pathophysiology. We had planned to deepen our understanding with an EUS biopsy of the mediastinal lymph nodes. Sadly, the patient's untimely demise precluded further exploration.

### 2.3. Treatment

The isolated *Aspergillus fumigatus* underwent susceptibility testing, which showed a minimum inhibitory concentration (MIC) level for voriconazole at 0.25 mcg/mL. Following the recommendations from the Infectious Diseases of America (IDSA) [8], the patient began a regimen of 150 mg of oral voriconazole, taken twice daily. Initiated on August 8, 2021, the voriconazole regimen continued until November 3, 2022. We also monitored her voriconazole levels periodically, which remained within the therapeutic range. Therapeutic drug monitoring confirmed that the voriconazole concentrations in her serum remained within the optimal therapeutic range. This monitoring is pivotal, as it can aid in adjusting dosages and identify potential drug interactions or adverse effects. Any existing contraindications or other medications the patient was receiving were also considered to ensure the maximum efficacy and safety of the voriconazole treatment.

## 3. Outcome and Follow-Up

The patient was initially discharged from the hospital with a plan for monitoring her health condition. However, she was readmitted shortly after because of the onset of hemoptysis. A subsequent bronchoscopy showed a reduction in granulation tissue and inflammation in the right bronchial tree. Following this, there was a noticeable improvement in her clinical condition. She was afebrile, had a significant decrease in her episodes of coughing, and reported alleviated dyspnea. Given her improved condition, she was deemed fit for discharge. Tragically, after her return home, she experienced a sudden onset of massive hemoptysis. By the time Emergency Medical Services (EMS) arrived at her residence, her condition had deteriorated rapidly. Immediate intubation was attempted on-site due to the severity of the hemoptysis, and cardiopulmonary resuscitation (CPR) was initiated. Despite their best efforts, the patient could not be resuscitated and was declared deceased. An autopsy, unfortunately, could not be conducted to ascertain further details.

## 4. Discussion

Cases of AT in people living with HIV were primarily documented between 1985 and 1996, with a significant decrease following the introduction of ART [9–11]. A recent case mirrored ours, involving a man living with HIV who also had neutropenia, presenting with symptoms similar to those of our patient [12]. Several factors that may increase the risk of AT include neutropenia either from a medical condition or from drugs affecting the bone marrow, use of corticosteroids, consumption of marijuana, previous episodes of *Pneumocystis carinii* pneumonia or cytomegalovirus disease, and taking broad-spectrum antibiotics, some of which are used as prophylaxis against *P. carinii* pneumonia. Compromised leukocyte function in individuals with HIV may also play a role. Chronic T-cell defects, which are seen in people with AIDS, might be involved, as T-cells often activate neutrophils and macrophages to display antifungal activity [3]. Nevertheless, since the introduction of ART, the number of people with HIV who develop aspergillosis has been dwindling [13]. Notably, in the past 2 years, there have been reports linking AT with COVID-19 infections [14, 15].

In our case study, the reason behind the development of aspergillosis tracheobronchitis in a person managing her HIV effectively remains puzzling. She showed a rare combination of symptoms including pseudomembranous tracheobronchitis and bronchial obstruction with granulation tissue. The progression of the disease in her suggests it started superficially, affecting the mucosa. This could hint at challenges in mucus production or ciliary function in individuals living with HIV, potentially evolving to ulceration and then to a more invasive stage. It is important to note that individuals with AT often experience milder immunosuppression than those with other forms of IPA [6]. The exact factors that make someone more susceptible to tracheobronchial infections rather than pulmonary parenchymal infections, especially in those with neutropenia or different immunosuppression levels, remain mostly undefined.

This case informs clinical practice by emphasizing that AT can occur even in patients with well-controlled HIV infection and without traditional risk factors. Clinicians should consider AT in the differential diagnosis when HIV-positive patients present with respiratory symptoms such as cough, fever, and dyspnea, regardless of their ART status or CD4 count. Early diagnostic procedures, including bronchoscopy and fungal cultures, are crucial for prompt identification and initiation of appropriate antifungal therapy, which can improve patient outcomes.

Furthermore, our observations suggest a testable hypothesis: individuals with well-controlled HIV infection may have localized immune dysfunction in the respiratory mucosa that predispose them to AT. Future research could explore mucociliary function and local immune responses in such patients compared to those without AT. Understanding these mechanisms might lead to targeted preventive strategies or treatments to reduce the risk of AT in this population.

The patient showcased an unexpected episode of severe hemoptysis, despite evident clinical improvement of the aspergillosis with voriconazole treatment. Such an occurrence has not been commonly reported in association with AT. It is worth emphasizing the inherently invasive nature of aspergillosis, particularly towards the vascular wall. This can give rise to vasculitis and the potential formation of infected aneurysms, a phenomenon that has been frequently observed in cerebral vessels [16]. Considering this, there's a plausible conjecture that a similar pathological mechanism might have transpired within the bronchial arteries of our patient. The propensity of IA for angioinvasion further underscores this hypothesis, providing a probable explanation for the sudden, severe hemoptysis. It is conceivable that, although the aspergillosis seemed well-managed, an infected aneurysm could have silently evolved and remained undetected in bronchoscopic evaluations. Furthermore, it is imperative to underscore that AT can lead to direct life-threatening complications, encompassing massive hemoptysis due to hyphal invasion into the pulmonary vasculature, acute airway obstruction from accumulated pseudomembranes of necrotic debris, and in some instances, such as in lung transplant recipients, the development of a bronchial-pulmonary artery fistula at the anastomotic site has been identified as a causative factor for fatal hemoptysis.

## 5. Conclusion

This case demonstrates that AT can occur in individuals with well-controlled HIV infection, even without traditional risk factors. Clinicians should consider AT in the differential diagnosis of respiratory symptoms in HIV-positive patients regardless of their ART status. Early diagnosis and prompt antifungal treatment are crucial for improving outcomes. The occurrence of fatal hemoptysis highlights the potential for severe complications, underscoring the need for vigilant monitoring even when clinical improvement is observed.

## Data Availability

The authors have nothing to report.

## Nomenclature

AIDS: Acquired immunodeficiency syndrome
ART: Antiretroviral therapy
AT: Aspergillus tracheobronchitis
BAL: Bronchoalveolar lavage
CD4: Cluster of differentiation 4 (a type of lymphocyte)
CPR: Cardiopulmonary resuscitation
CT: Computed tomography
EMS: Emergency Medical Services
GMS: Gomori methenamine silver (stain)
GM: Galactomannan
HIV: Human immunodeficiency virus
IPA: Invasive pulmonary aspergillosis
LDH: Lactate dehydrogenase
MIC: Minimum inhibitory concentration
ODI: Optical density index
PCR: Polymerase chain reaction
PI: Protease inhibitor
RML: Right middle lobe
RUL: Right upper lobe
TB: Tuberculosis
